# Pronator quadratus repair after volar plating of distal radius fractures or not? Results of a prospective randomized trial

**DOI:** 10.1186/s40001-015-0187-4

**Published:** 2015-11-25

**Authors:** Sandra Häberle, Gunther Hubertus Sandmann, Stephan Deiler, Tobias Maximilian Kraus, Florian Fensky, Tobias Torsiglieri, Ina-Christine Rondak, Peter Biberthaler, Ulrich Stöckle, Sebastian Siebenlist

**Affiliations:** Department of Trauma Surgery, Klinikum Rechts der Isar, Technische Universität München, Ismaninger Str. 22, 81675 Munich, Germany; BG Trauma Center, Eberhard Karls University, Schnarrenbergerstr. 95, 72076 Tübingen, Germany; Department of Trauma Surgery, University Medical Center Hamburg-Eppendorf, Martinistr. 52, 20246 Hamburg, Germany; Department of Oral, Maxillofacial and Plastic Facial Surgery, RWTH Aachen University Hospital, Templergraben 55, 52062 Aachen, Germany; Institute of Medical Statistics and Epidemiology, Klinikum Rechts der Isar, Technische Universität München, Ismaninger Str. 22, 81675 Munich, Germany

**Keywords:** Pronator quadratus, Distal radius fracture, Pronation strength, Volar plate, Pronator quadratus repair

## Abstract

**Background:**

The purpose of the present study was to investigate the influence of the pronator quadratus (PQ) muscle repair following volar plate fixation of distal radius fractures with special regards to the forearm pronation strength. During the early recovery period of 3 months, an improvement of pronation strength and functional scorings was hypothesized for the PQ repair when compared to no repair.

**Methods:**

The inclusion criteria were (1) men or women between 18 and 80 years, (2) isolated, closed fractures of the distal radius, (3) A2 to B2 types of fracture according to the AO fracture classification system, (4) primary volar locking plate osteosynthesis. Patients were randomized to group A = PQ repair and group B = no repair. Follow-up examinations after 6 and 12 weeks included bilateral isometric pronation strength measurement, range of motion, the *Quick*DASH and the Mayo-Wrist-Score, and a visual analog scale (VAS).

**Results:**

60 patients (*n* = 31 in group A and *n* = 29 in group B) with an average age of 54 years (range 22–77 years) returned for both follow-up visits. The pronation strength measurements showed no significant differences between groups (PQ repair vs. no repair) neither at 6 weeks nor at 12 weeks. Additionally, no statistical significant differences were noted for ROM, *Quick*DASH-Score or Mayo-Wrist-Score. The VAS scoring revealed a significant decreased pain level after PQ repair at 6 weeks postoperatively (*p* = 0.017).

**Conclusion:**

An improved pronation strength after PQ repair in the early rehabilitation period could not be confirmed. However, the PQ repair might reduce pain in the early postoperative period.

Trial registration number: NCT02595229 (ClinicalTrials.gov, registered 02 November 2015)

## Background

Distal radius fractures account for the most common fractures (up to 25 %) and their incidence is increasing in an aging population [1, 2]. The volar locking plate osteosynthesis has become the treatment of choice for distal radius fractures in recent years. This technique enables good functional results with high fragment stability and a lower complication rate than alternative procedures as external fixation, closed reduction and casting, percutaneous pin fixation or dorsal plating [3, 4]. During the volar approach the release of the pronator quadratus muscle (PQ) from its radial insertion is required for visualization and fixation of the fracture at the distal radius [5, 6]. In this context, the suturing of the PQ muscle following plate fixation is controversially discussed. According to the current literature the assumed benefits of this muscle repair include restoration of the pronation strength, protection of the flexor tendons, and stability of the distal radioulnar joint [7–9]. However, a sufficient repair of the PQ muscle can often be difficult due to the poor tissue quality [10]. It is described that a tight repair might even lead to ischemic contractures with a subsequently decreased range of motion [11]. A number of studies evaluated the efficacy of PQ repair in patients with repair of the PQ muscle versus no repair and reported no differences in terms of functional outcome [12, 13]. However, these studies lacked objective strength testing in forearm pronation.

The main purpose of this prospective trial was to determine the influence of PQ repair on forearm pronation strengths during the initial rehabilitation period of 3 months after surgery. The hypothesis was that a repair of the PQ muscle after volar plating of distal radius fractures leads to higher isometric pronation strengths when compared to no PQ repair. Moreover, an improved functional outcome based on validated upper extremity scores was assumed with PQ repair following volar plate osteosynthesis.

## Methods

### Study population

The present survey was performed as a prospective, randomized controlled single-center study. The local institutional ethics committee approved the study protocol (study number 2759/10), trial registration number NCT02595229 (ClinicalTrials.gov, registered 02 November 2015). From May 2010 to March 2013, 72 consecutive patients were included in the study. The inclusion criteria were (1) male or female patient with an age between 18 and 80 years, (2) isolated, closed fractures of the distal radius, (3) A2 to B2 types of fracture according to the AO fracture classification system (4), primary volar locking plate osteosynthesis within 7 days after trauma. For exclusion the following criteria were determined: (1) concomitant fractures of the affected upper extremity, (2) intra-articular distal radius fractures (type C), (3) concomitant neurovascular injuries or preexisting neurological illnesses, (4) initial external fixation. The study was performed as a single-blinded, controlled randomized trial. Randomization into group A = repair of the PQ muscle and group B = without PQ repair was performed by an independent member of the institutional study center. Written informed consent was obtained from each patient when initially presented at the emergency room.

### Surgical procedure and postoperative management

**Fig. 1:**
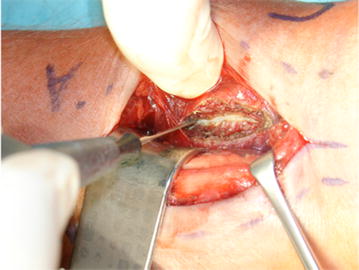
Incision of the PQ muscle at the radial third of the distal radius

All operations were performed by experienced upper extremity surgeons under regional or general anaesthesia. Following standard volar approach, a complete longitudinal incision of the PQ muscle at the radial third with a subsequent careful elevation of the muscle from the bone was performed for visualization of the distal radius in all cases (Fig. 1).

After fracture reduction and consecutive volar plate fixation, in group A the PQ muscle was sutured with three to five U-shaped stitches using a polyfilament, absorbable synthetic suture (3.0 Vicryl, Ethicon Norderstedt, Germany). In the control group (group B), the PQ was just placed to its anatomic position but without suture repair. For plate osteosynthesis, in three cases a 2.4 mm unidirectional locking plate (Synthes, Umkirch, Germany) was used. The remaining included patients had received a 2.5 mm multidirectional fixed-angle plate (Distal Radius 2.5, Medartis, Basel, Switzerland).

All the patients underwent the same postoperative management irrespective of the study arm. Within the first 2 weeks the wrist was immobilized in a cast for active motion. Passive exercises started under physiotherapist’s supervision the day after surgery. After 4 weeks the cast was removed and physiotherapy with unrestricted active range of motion was allowed. Weight bearing was restricted for 6 weeks postoperatively.

### Outcome parameters

Patients returned 6 and 12 weeks after surgery for follow-up visits. Personal interviews and physical examinations were carried out by an independent investigator (SH) not involved in the initial surgical procedure.

**Fig. 2:**
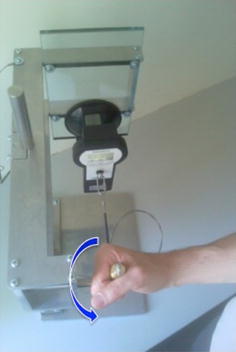
Setup for pronation strength measurement: the *blue arrow* indicates the isometric pronation directional movement

As primary outcome parameter the isometric pronation strength was measured using the IsoForce-Control EVO 2 dynamometer (MDS AG, Oberburg, Switzerland) on a self-built measurement apparatus (Fig. 2). With the flexed forearm (in 90°) in neutral position, the operated arm was tested with three isometric effort trials (5 s/trial) while recording the maximum and mean pronation strength of every trial. The averaged values were used for evaluation.

Secondary outcome parameters included the range of motion (ROM) of both wrists, the shortened Disabilities of the Arm, Shoulder and Hand questionnaire (*Quick*DASH), the Mayo-Wrist-Score, and a visual analog scale (VAS) (range 0 points = no pain to 10 points = maximum pain) [14–16].

Moreover, postoperative radiographs were reviewed for maintenance of fracture reduction and implant-related complications.

### Statistical analysis

Continuous variables are presented as mean ± standard deviation (SD) or median and range in case of skewed distributions. Categorical variables are presented as absolute and relative frequencies. Differences between groups were assessed using a two sample *t* test, a Mann–Whitney *U* test, or a Fisher’s exact test, as appropriate. For the comparison of different time points within a group, paired t test or Wilcoxon signed rank test was used. All reported *P* values are two sided, with a significance level of 0.05 and have not been adjusted for multiple testing. Statistical analysis was performed using IBM SPSS Statistics for Windows, version 22 (Armonk, NY, USA: IBM Corp.).

## Results

From the 72 included patients, 60 patients (47 females, 13 males) with an average age of 54 years (range 22–77 years) returned for both follow-up visits.

31 patients (group A) with a mean age of 52 years (range 22–77 years; 24 females and 7 males) had a PQ repair, whereas 29 patients (group B) with a mean age of 56 years (range 25–76 years; 24 females and 5 males) had no PQ muscle repair. In both groups all fractures were A-type distal radius fractures according to the AO classification. There were two A2-type and 29 A3-type fractures in group A, and five A2-type and 25 A3-type fractures in group B, respectively.

Two patients dropped out of the study. One patient (group B) needed to be revised due to loss of fracture reduction 15 days after index surgery and another patient (group A) has developed a carpal tunnel syndrome 6 weeks postoperatively and resulted in reoperation. 10 patients were not available for both follow-up examinations and were, therefore, secondarily excluded.

### Isometric pronation strength

**Fig. 3:**
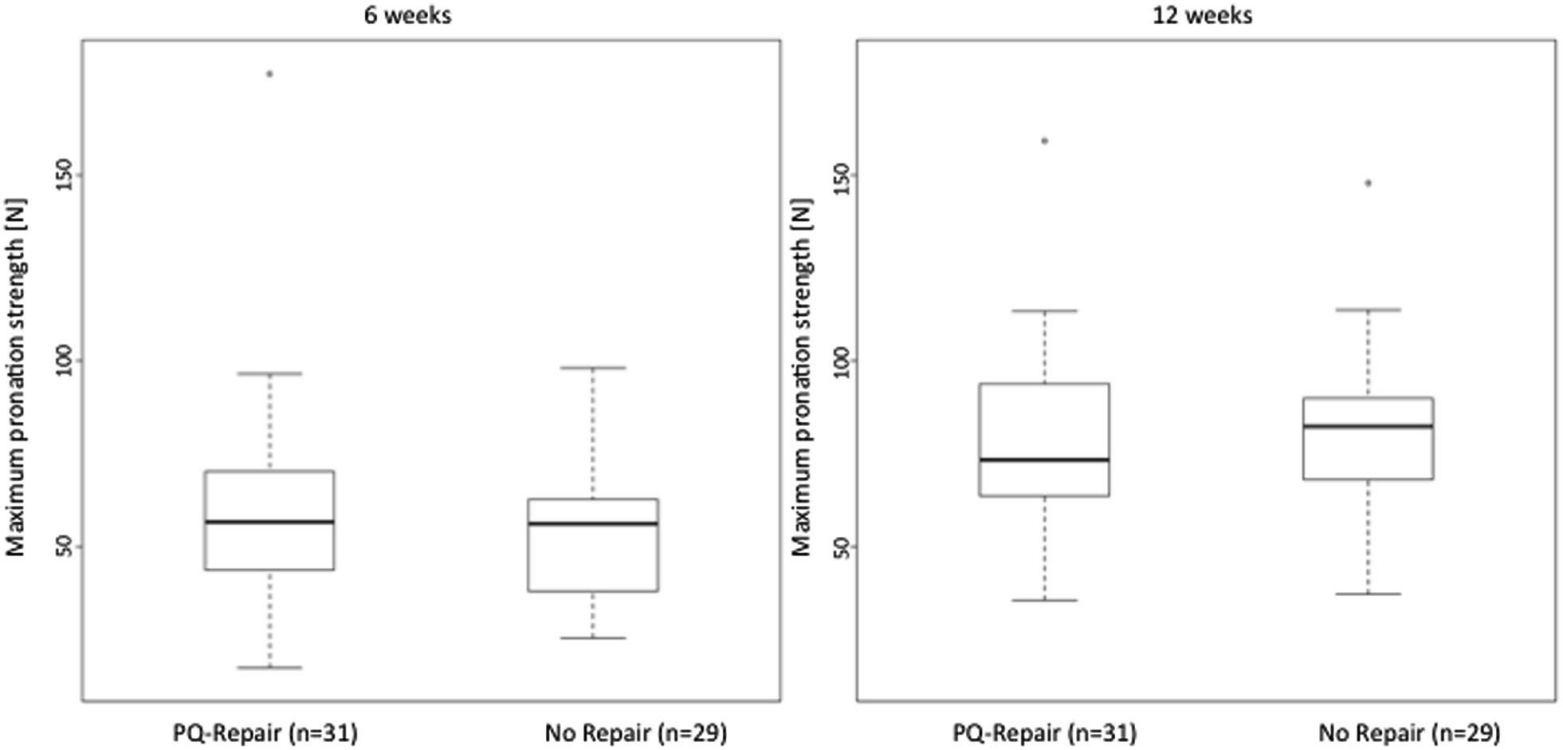
Maximum pronation strength (*F* ^max^)

**Fig. 4:**
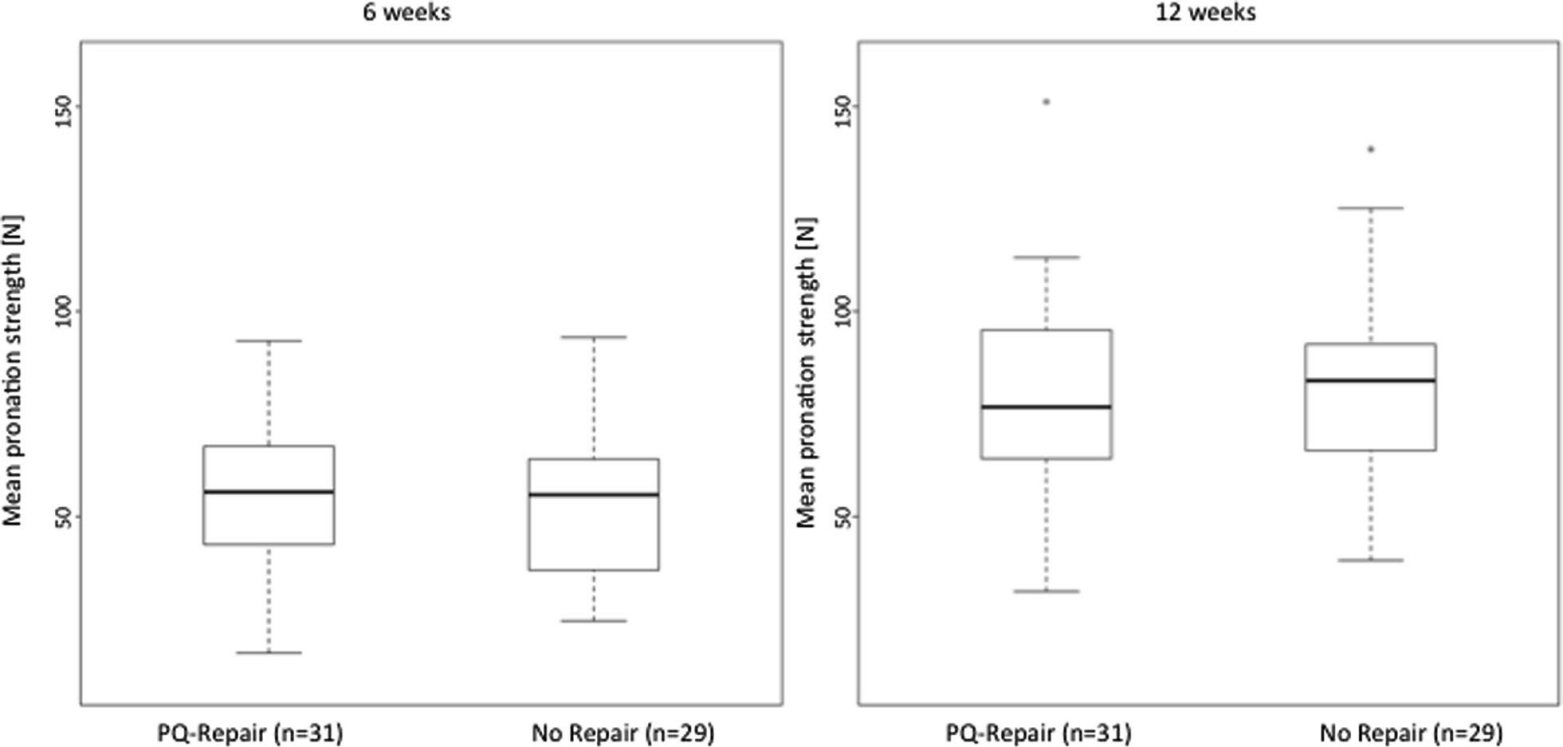
Mean pronation strength (*F* ^mean^)

At 6 weeks postoperatively, higher values for the PQ repair (±30 %) were seen for the maximum pronation strength (*F*^max^) but without significant differences between groups [group A—median 52.1 N (range 22.0–232.9 N); group B—median 40.2 N (range 23.1–92.0 N); *p* = 0.112]. Accordingly, the median of the mean pronation strength (*F*^mean^) was 44.8 N (range 19.4–172.7 N) for group A (±19.5 %) and 37.5 N (range 21.2–82.6 N) in group B (*p* = 0.169) (Figs. 3, 4). For further analysis, the ratio (Δ*F*) between the operated and non-operated hand was evaluated for maximum and medium strengths. There could also not be found any significant differences between group A and B (for *F*^max^*p* = 0.599 and for *F*^mean^*p* = 0.610).

12 weeks after surgery, increased pronation strengths were found in both groups with *F*^max^ of median 65.9 N (range 28.5–210.0 N) in group A (±7.9 %) and 61.1 N (range 38.3–123.4 N) in group B, but without statistical significance between groups (*p* = 0.333). The median of *F*^mean^ was 63.6 N (range 27.2–203.3 N) in group A (±7.8 %) and 59.0 N (range 35.3–111.8 N) in group B (*p* = 0.403) (see Figs. 3, 4).

Similar to the 6-week follow-up, at 3 months postoperatively no significant differences were detected for the group comparison of Δ*F* (for *F*^max^*p* = 0.641; for *F*^mean^*p* = 0.403).

### ROM

The results of ROM are summarized in Table 1. Neither at 6 weeks nor at 12 weeks after surgery any differences were observed between groups. Furthermore, for each group no statistically significant differences in ROM were seen when confronted the injured with the uninjured side (not shown).

**Table 1:** ROM after 6 and 12 weeks (*EF* extension–flexion arc, *PS* pronation–supination arc, *RU* radial abduction–ulnar abduction arc), values are depicted in median (min–max)

	6 weeks			12 weeks		
	PQ repair (*n* = 31)	No repair (*n* = 29)	*P* value	PQ repair (*n* = 31)	No repair (*n* = 29)	*P* value
EF	75° (40–150)	90° (20–160)	0.332	120° (60–180)	130° (40–180)	0.441
PS	170° (120–180)	160° (50–180)	0.073	180° (160–180)	180° (160–180)	0.333
RU	50° (25–70)	50° (15–80)	0.200	60° (30–110)	60° (25–90)	0.525

### Outcome scoring

**Fig. 5:**
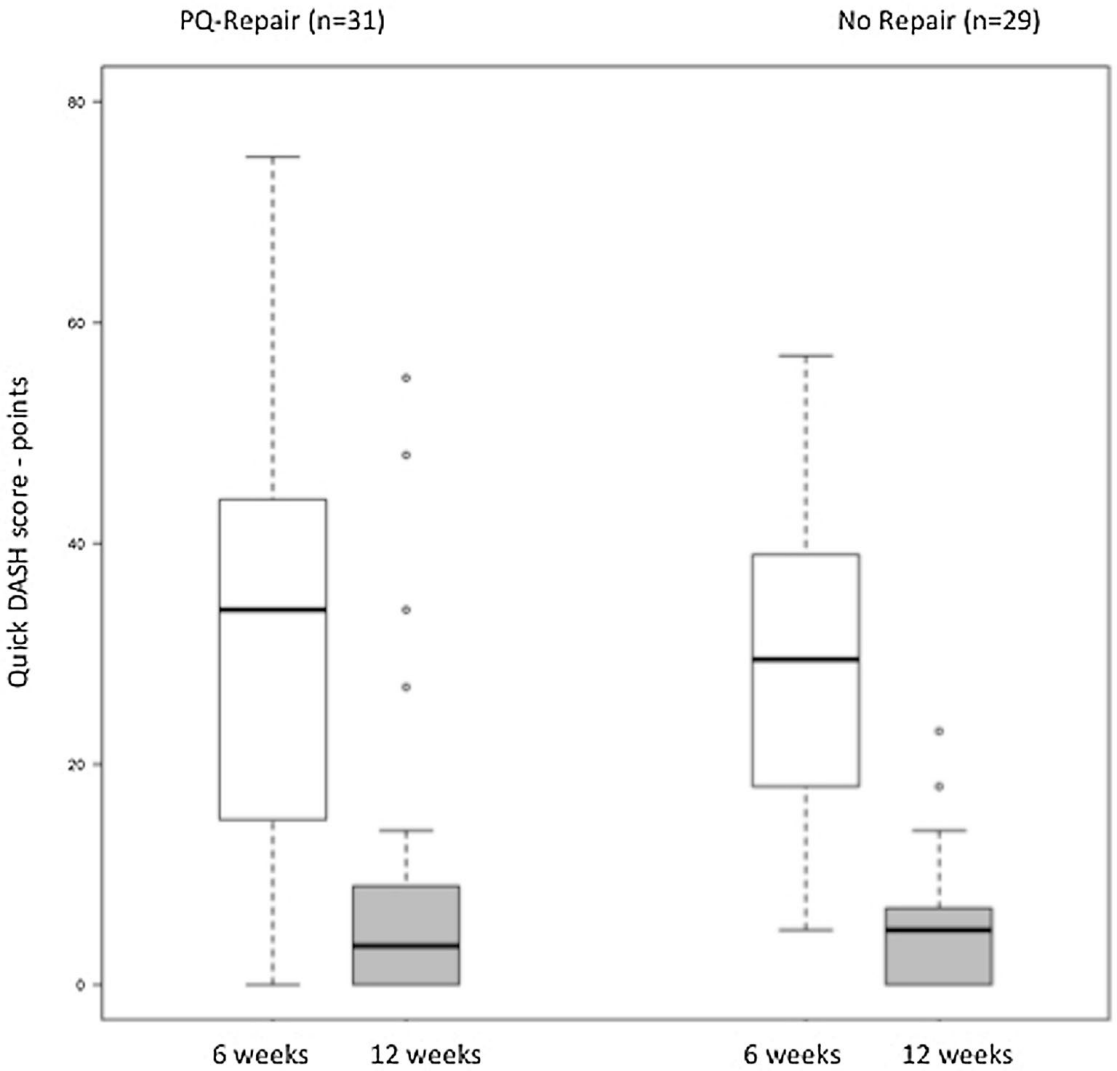
QuickDASH at 6 and 12 weeks after surgery

At 6 weeks postoperatively, the *Quick*DASH-Score showed a median of 34 (range 0–75) points in group A and 30 (range 5–57) points in group B (*p* = 0.789). After 12 weeks, the values strongly decreased with a *Quick*DASH of 3.5 (range 0–55) points in group A and 5 (range 0–23) points in group B indicating an improved functional outcome (*p* = 0.887) (Fig. 5).

For the Mayo-Wrist-Score, satisfying results were shown after 6 weeks in both groups with a median of 70 (range 30–100) points in group A (including 8 excellent, 6 good, 7 satisfying, and 10 bad outcomes), and 70 (range 50–110) in group B (including 1 excellent, 8 good, 13 satisfying, and 7 bad outcomes). At 12 weeks the values improved in both groups representing excellent results with median 100 (range 40–100) points in group A (25 excellent, 4 good, 2 bad outcomes) and 95 (range 50–100) points in group B (23× excellent outcomes, 5× good outcomes, 1× bad outcome). No significant differences could be verified between groups (*p* = 0.994 at 6 weeks; *p* = 0.657 at 12 weeks).

### VAS

**Fig. 6:**
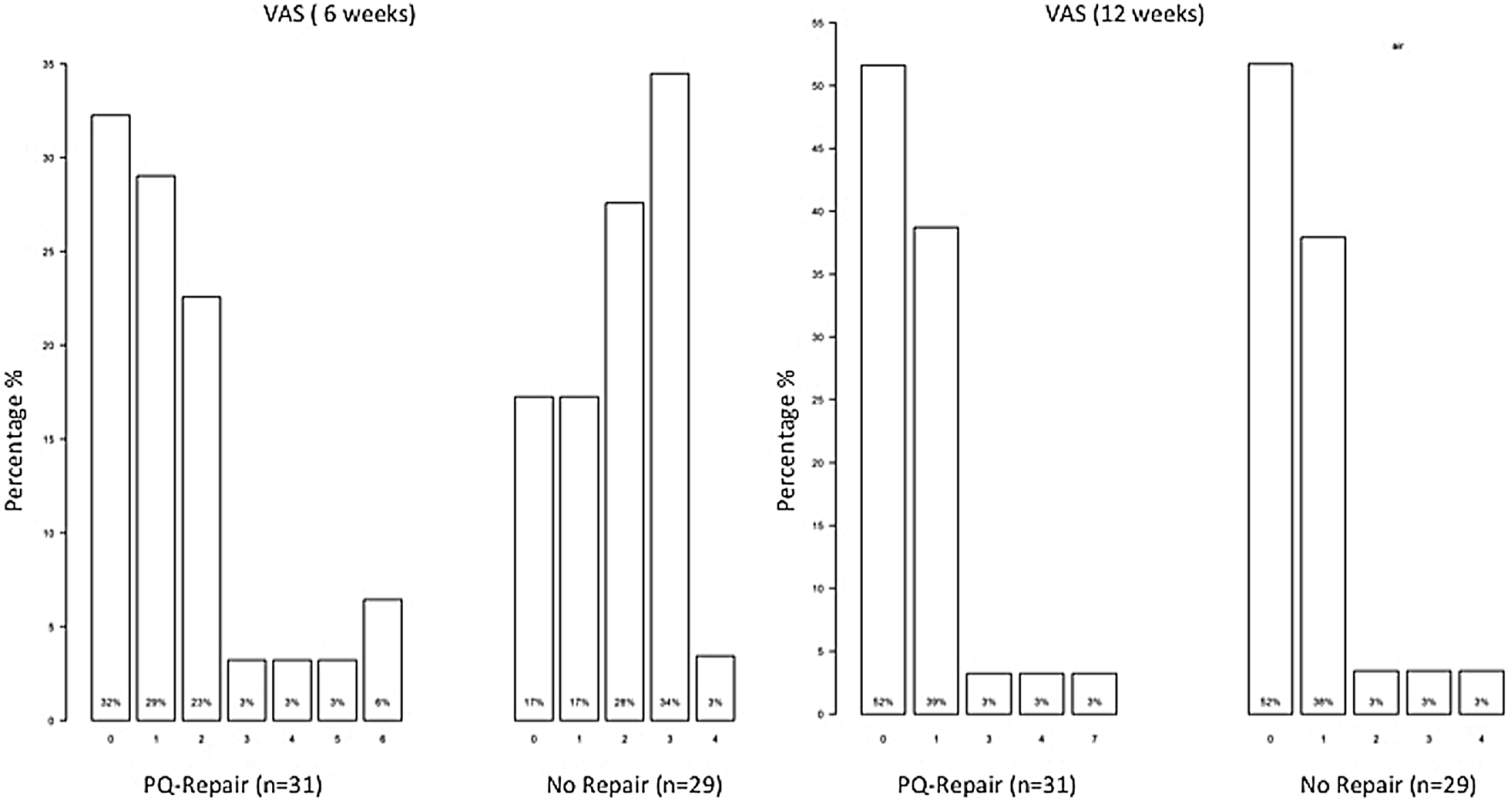
VAS values at 6 and 12 weeks after surgery

At 6 weeks postoperatively a significant reduction of pain was found following repair of the PQ muscle with a pain level between 0 and 2 in 84 % of the patients in group A versus only 62 % in group B (*p* = 0.017). At the 12-week follow-up no significant difference could be seen (pain level between 0 and 2 in group A—91 % and group B—93 %; *p* = 1.000) (Fig. 6).

For both groups, complete bony healing was seen in all fractures after 12 weeks without any implant-related complications. There were no wound healing disturbances and tendon irritations or ruptures, respectively.

## Discussion

The pronator quadratus muscle repair after volar plating of distal radius fractures is still a topic of debate in the current literature. According to a recent survey, the majority of surgeons attempt to repair the PQ muscle after plate fixation [17]. Even though there are several studies evaluating the functional outcome with special respect to the PQ repair, to the best of our knowledge, no prospective comparative study measuring the objective pronation strength has been published to date [4, 12, 13, 18, 19]. The aim of the present study was to prove the theory that a PQ muscle repair leads to higher isometric pronation strengths in the initial postoperative period. Moreover, various functional outcome parameters were assessed to be compared between patients with PQ repair versus no repair.

The most important finding of this study was that an increased pronation strength in patients with PQ repair was evident 6 and 12 weeks after surgery. Nevertheless, the higher values of maximum (+30 %) at 6 weeks; (+7.9 %) at 12 weeks and mean (+19.5 %) at 6 weeks; (+7.8 %) at 12 weeks pronation strengths in the PQ repair group were not statistically significant compared to the no repair group, so that the aforementioned hypothesis could not be confirmed. These strength deficits, however, match favorably to those of the study of McConkey et al. [20]. The authors reported on a 16–23 % significant loss of isometric pronation torque (depending on forearm rotation) upon complete denervation of the PQ muscle in healthy volunteers when compared to the normal arm side, and these results likely correlate with those of patients without PQ repair in this study. The decreasing difference in pronation strength between groups during the follow-up visits is probably caused by muscle and soft tissue scarring, regardless of a PQ repair or not. The question, however, remains whether a difference of approximate 7.9 % in pronation strength is clinically relevant in the rehabilitation period 12 weeks after surgery or later. For answering this question, further evaluations with a longer follow-up are needed.

Furthermore, in the present study, groups were confronted with respect to the strength ratio (Δ*F*) between the operated and non-operated armside. The rationale, therefore, was that it seems natural that a straight comparison of the affected and non-affected extremity in the early postoperative period would show decreased strength values. Nevertheless, no differences were found between groups either. Huh et al. demonstrate less isokinetic pronation strengths at 6 months in the operated forearm than in the normal side, but at 12 months postoperatively, the differences in pronation strength were not significant anymore [19]. As consequence the authors suggested that dissection of the PQ may have minimal clinical impact on the forearm pronation function. Nonetheless, the authors also pointed out that dissected PQ muscles were not completely repaired in their cohort of patients and assumed a better pronation power during the early recovery period with a preserved or well-repaired PQ muscle [18]. This fact could not be verified in the present study.

High functional outcomes (near preinjury ROM, minor values of the *Quick*DASH indicating a very low level of upper extremity disability, excellent results of the Mayo-Wrist-Score in >93 % of the patients) were seen at the latest follow-up in both groups, even though without differences among each other. Trosti and Ilyas [13] also performed a prospective evaluation of pronator quadratus repair versus no repair following volar plate fixation with a minimum follow-up of 12 months. They even found no significantly different results between groups according to ROM at the wrist, DASH scores, grip strength, and VAS scores. These findings as well as the results of the present study compare favorably to those of other recently published studies [21, 22]. In a retrospective study with a 3-month follow-up, Ahsan et al. [4] found no differences in ROM and grip strength in 108 patients with complete and incomplete PQ repair. Hershman et al. [12] examined the outcome effects in 112 patients treated with or without PQ repair after a follow-up of 1 year. They found no differences in pronation, pain and DASH scores and, therefore, concluded that there is no advantage in repairing the PQ muscle during volar plating of distal radius fractures.

The lack of significant differences between groups in the present study may be caused by PQ repair failure as well. Nevertheless, Swigart et al. [17] in their prospective clinical cohort study have shown that only 4 % of the PQ repairs failed within the first 3 months after surgery. The repair of the pronator quadratus muscle, however, can be frequently challenging due to various reasons such as traumatic disruption, fracture comminution or poor soft tissue quality, particularly in aged patients. For that reason patients presenting with open fractures and complex intra-articular fracture types were excluded from the present study. In the repair group the reconstruction of the PQ muscle could be performed in all patients. There are some few authors who state that a complete or, if not possible, at least an incomplete PQ repair is the critical factor to reduce flexor tendon irritations or ruptures [4, 23]. In recent years, diverse authors have, therefore, reported on pronator sparing or splitting techniques to address this difficulty [10, 24]. The reported incidence of tendon complications after volar plating varies between 2 and 12 % [11, 22, 25, 26]. As opposed to this, in both groups of the present study no tendon irritations or ruptures were clinically apparent during the follow-up period and similar results were also shown in the current literature [4]. Correspondingly, a significant difference in pain (measured on VAS scale 0–10) was observed between the PQ repair and no repair group (*p* = 0.017) within the initial rehabilitation period of 6 weeks postoperatively. This fact might be explained by the better hardware coverage and, therefore, less irritation of the overlying flexor tendons.

This study has some limitations. First of all, it has to be clearly stated that the results seen in this very short duration of follow-up might change with a longer follow-up term. However, concerning about the functional outcomes of cited studies above that have shown no differences up to 1 year of follow-up, it is doubtful whether the pronation strength or other functional parameters will be changed during the further postoperative time period [4, 12, 18, 19]. This study did not examine pronation strengths in relation to the dominant arm side, which can potentially be influenced by a PQ repair or not, and moreover, the quality of fracture reduction or plate positioning was ignored. A power analysis has not been performed a priori, as, during the planning of the study, no comparable studies have been published.

Nevertheless, this study has several strengths presented by multi-functional outcome scorings including objective pronation strength testing of all included patients at both follow-up visits and the comparability of evaluated groups. The demographic characteristics (age, gender) as well as the fracture pattern and the used implants were matchable between both groups. Furthermore to our research of the literature, this study is the first prospective randomized trial evaluating the objective forearm pronation strengths after volar plating of distal radius fractures and pronator quadratus muscle repair.

## Conclusions

In the present study, with the PQ muscle repair after volar locked plating of distal radius fractures only a positive trend could be shown on forearm pronation strength in the early postoperative period of 3 months. The statement, however, that a PQ repair will lead to a higher pronation strength versus no repair could not be proven. The authors, therefore, conclude that there is no advantage in repairing the PQ muscle during volar plating of distal radius fractures in terms of pronation strength improvement. Even though not supported by the present data, the authors subscribe to previous authors’ recommendation for a careful reconstruction of the PQ muscle to reduce postoperative pain, achieve safe coverage of the implant and thus potentially avoid postoperative complications.

## Abbreviations

PQ: pronator quadratus muscle
VAS: visual analog scale
ROM: range of motion
*F*^max^: maximum pronation strength
*F*^mean^: mean pronation strength
